# Shocked to death: a case report of cardiogenic shock and death following electrocardioversion for atrial fibrillation

**DOI:** 10.1186/s12872-023-03376-8

**Published:** 2023-07-14

**Authors:** Anas Mohamad Hashem, Omar Al Ali, Amani Khalouf, Ahmed Shehadah, Moghniuddin Mohammed, Amir Mahmoud, Maryrose Laguio-Vila, Mohan Rao

**Affiliations:** 1 Internal medicine resident, Rochester General Hospital, Rochester, NY 14621 USA; 2 Sands-Constellation Heart Institute, Rochester General Hospital, Rochester, NY 14621 USA; 3 Department of Infectious Disease, Rochester General Hospital, Rochester, NY 14621 USA

**Keywords:** Electrocardioversion, Atrial fibrillation, Cardiogenic shock, Case Report

## Abstract

**Background:**

Atrial fibrillation (AF) is prevalent, especially in patients with heart failure. Their prevalence increases with age and both conditions are interrelated. Electrocardioversion (ECV) is considered a safe and effective procedure and is among one of the recommended therapies to terminate AF back to normal sinus rhythm. Our study highlights one of the rare complications following ECV.

**Case summary:**

A 71-year-old female with a history of atrial fibrillation underwent electrocardioversion and developed sudden onset of ventricular stunning resulting in refractory cardiogenic shock. She was treated with mechanical cardiac support including IABP and Impella. Both provided minimal support then rapid clinical deterioration happened leading to imminent death.

**Conclusion:**

Patients with atrial fibrillation and heart failure treated with electrocardioversion might develop refractory cardiogenic shock and death as a complication of this procedure.

**Supplementary Information:**

The online version contains supplementary material available at 10.1186/s12872-023-03376-8.

## Introduction

AF has been more prevalent in the United States given the growth of the older population. In 2010, around 5.2 million patients had AF and it was estimated to reach up to 12.1 million by 2030 [1]. In addition, HF is present in 6 million American adults in the same census, and likewise, its prevalence increases with age [1]. According to Ruddox et al., AF and HF are interrelated, and patients with AF are at nearly 5-folds increased risk for developing HF [2]. The Framingham Heart Study revealed that incidence of HF in patients with AF increased all-cause mortality and was associated with a worse prognosis [3]. Thus, treatment of AF in HF must be prompt to improve the mortality. ECV is a recommended option to terminate AF back to NSR and is considered to be a safe and effective procedure with minimal AE [4]. Interestingly, we report a case with AF and HF who underwent ECV and developed LV stunning complicated with cardiogenic shock, and inevitable death.

## Case presentation

### History of presentation and physical examination

A 71-year-old female with a history of AF on anticoagulation complicated by tachycardia-induced cardiomyopathy and systolic HF (baseline EF of 45%) presented to the emergency due to a sudden onset of dyspnea and palpitations. Her vital signs were initially stable with BP = 112/64 and HR = 150. On physical examination, she had jugular venous distension, irregular heart rhythm with a rate of 150bmp, and reduced air entry bilaterally with crackles at the bases of the lung. Also, she had bilateral pitting edema.

### Past medical history

She has a history of diabetes mellitus, hypertension, hyperlipidemia, rheumatic fever as a child, moderate mitral regurgitation without stenosis, AF, and HF with reduced EF. She was diagnosed with AF in 2014 and was placed on metoprolol and Rivaroxaban. 2 months prior to her current hospitalization, she underwent cardioversion which temporarily terminated AF. A month later, patient elected to undergo catheter ablation instead being placed on antiarrhythmics, therefore, she had catheter ablation for a recurrent AF which was able to revert her heart rhythm back to NSR. Her ambulatory transesophageal echocardiogram showed: The left ventricular cavity size is normal (LVIDd/BSA = 2.8 cm/m^2^), with normal mass (LV mass index = 83.3 g/m^2^), with mildly reduced systolic function EF = 45%. There is mild global hypokinesis of the left ventricle. The mitral valve leaflets appear thickened, but open well, with no valvular stenosis, but moderate amount of mitral regurgitation. By PISA method, the effective regurgitant orifice area (ERO) is 0.19 cm [2] and volume (RV) is 22.3 ml.

### Differential diagnosis

Her symptoms were presumed to be either acute decompensated HF, acute mitral regurgitation, or related to recurrence of AF with RVR complicated by tachycardia-induced cardiomyopathy.

### Diagnostic assessment

**Fig. 1:**
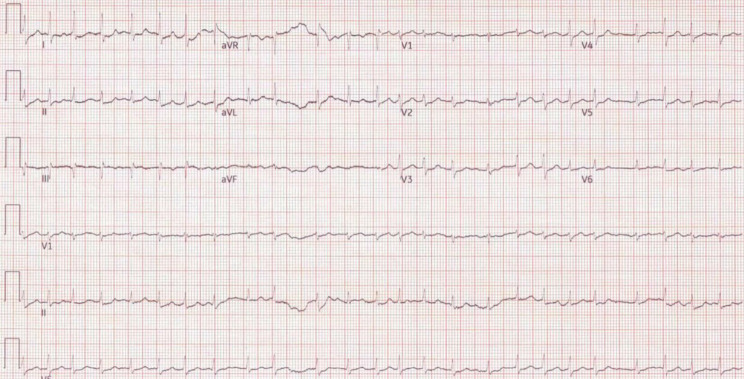
Baseline electrocardiogram (EKG) upon admission revealing atrial fibrillation with a HR of 150 beats/min

**Fig. 2:**
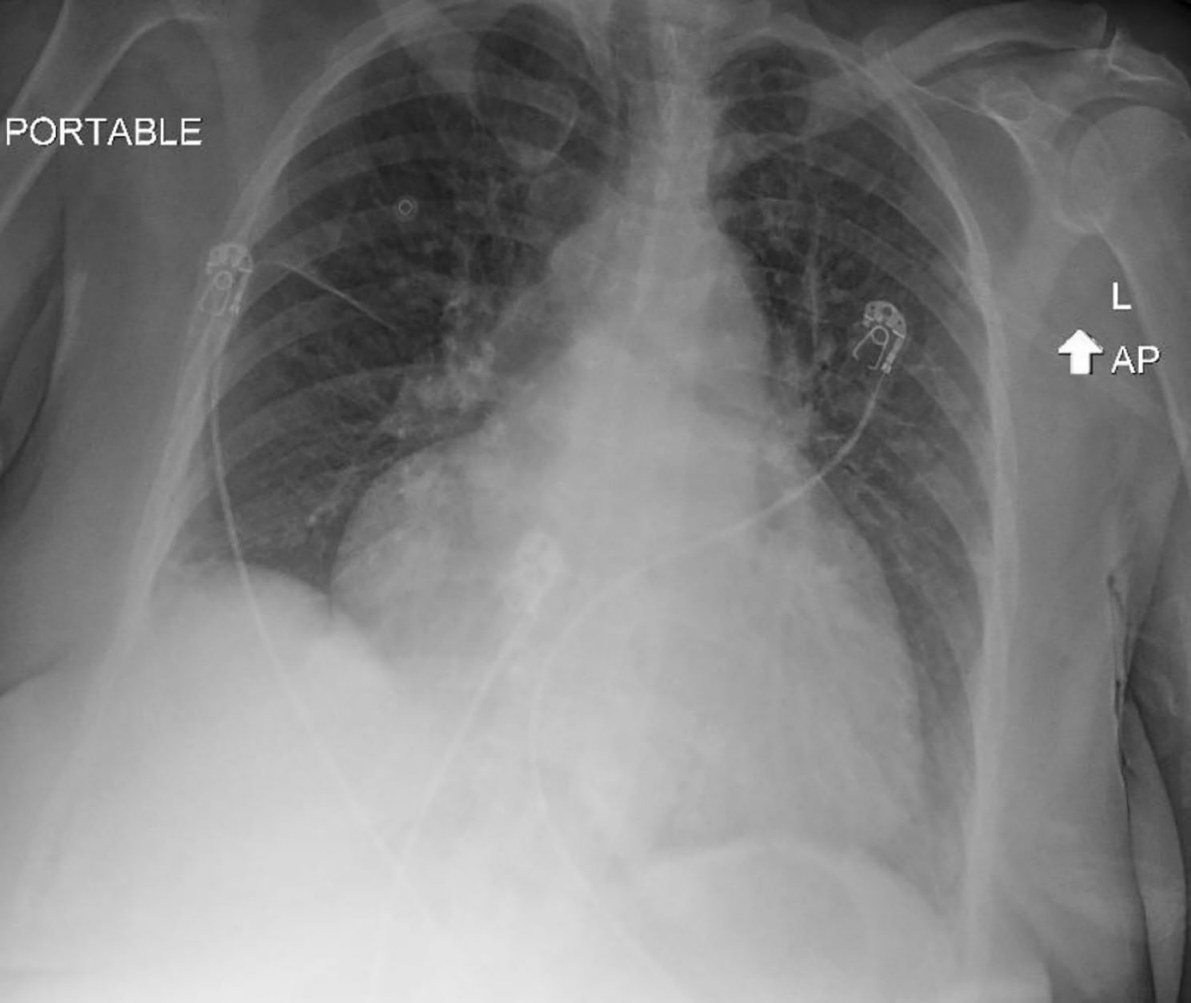
Chest x-ray upon admission showing an enlarged heart with an increase in the cardiothoracic ratio. No visible obliteration of the costophrenic angles

An urgent EKG revealed AF with RVR (150 bpm) with a QTc of 410 msec (Fig. 1). Chest x-ray showed moderate bilateral pulmonary congestion with new enlargement of the cardiac silhouette suggestive of secondary cardiomegaly or pericardial effusion (Fig. 2). Lab investigations showed WBC of 8.9, Hb of 9.5, serum chemistry, elevated liver enzymes (AST = 382U/L and ALT = 395U/L), high-sensitivity troponin I at baseline was 174 and 180 after an hour with a delta of 6 (N = 0–9), and elevated BNP 856 pg/mL.

### Interventions and follow up

**Fig. 3:**
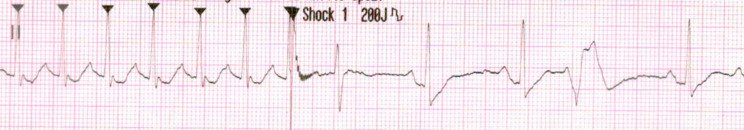
Cardiac rhythm during cardioversion on telemetry

**Fig. 4:**
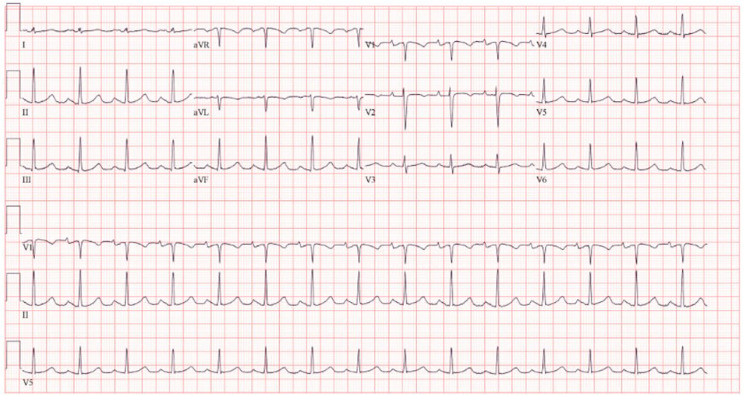
Following electrocardioversion, EKG showing reversion of the patient?s heart rhythm back to normal sinus rhythm 90 beats/min

To symptomatically manage her, she received a dose of 60 mg IV furosemide with minimal improvement. As she continued to be symptomatic in the setting of AF with RVR (HR = 148 bpm, BP = 109/83mmHg), we attempted a dose of 5 mg IV metoprolol without any response, then 150 mg bolus of IV Amiodarone followed by an amiodarone infusion (1 mg/min) for 8 h and it was not successful in improving her HR with worsening of her symptoms. Therefore, an urgent cardioversion was planned (HR = 152, BP = 113/53mmHg). A quick pre-interventional bedside TTE revealed presence of a small pericardial effusion without tamponade activity, global mild left ventricular hypokinesis with mildly reduced systolic function (EF = 40–45%) and ruled out right ventricular dysfunction. A synchronized ECV at 200 J was performed and was successful in restoring NSR (HR = 90 bpm, BP = 114/78mmHg), Figs. 3 and 4. Despite being in NSR with a QTc of 397 msec, she immediately developed hypotension after CV which was refractory to fluids and pressors. Urgent bedside echocardiogram revealed severe global hypokinesis (EF = 10–15%) with ventricular stunning. She subsequently went into cardiac arrest with PEA and achieved ROSC after 4 min of CPR. She was emergently taken for left and right heart catheterization which showed normal coronary vessels with moderate PHTN = 56/30(42) mmHg, elevated wedge pressure = 35, CO/CI was low (2.38/1.32) and SVR was elevated (1,613), which goes in favor of cardiogenic shock. Given her severely reduced CI, decision was made to provide mechanical cardiac support with an IABP. Initially, her BP was maintained around 120s/70s with an augmentation of 127mmHg, however, after 20 min, her CI and BP dropped and reached down to 1.27 and 46/19mmHg, respectively. Therefore, it was advanced to Impella 2.5 with a flow of 3.2 L/min and her CI went up to 4.3, Additional File Video 1. Echocardiogram following percutaneous heart pump revealed persistence of global hypokinesis of the left ventricle with normal ventricular size and an EF of 20%, with moderate mitral valve regurgitation, similar to her ambulatory TTE. The possibility for Takotsubo-syndrome was less likely given the diffuse hypokinetic involvement. The next day, she was given 160 mg IV furosemide, followed by 120 mg and then was placed on a furosemide drip (30 mg/hr) for 2 days. She improved transiently but continued to have poor perfusion and despite maximal support with norepinephrine, phenylephrine, vasopressin and epinephrine drips, she developed multiorgan failure. Her condition and vitals further deteriorated despite maximum efforts, a goal of care discussion was conducted with her family, and they pursued comfort care. Pressors and Impella device were stopped, and patient ultimately passed away. An autopsy was offered for further evaluation, but it was repudiated by her family.

## Discussion

To our knowledge, this is the first study to report a case with AF complicated by AIC that was treated with ECV and developed immediate refractory cardiogenic shock unresponsive to mechanical cardiac support including IABP and Impella.

Atrial fibrillation has been associated with an increased risk for heart failure incidence and presence of both condition leads to worse prognosis [5]. Evidence has shown that AF with/without HF is associated with structural, neurohumoral and electrophysiological changes [6]. Aggressive approach in treating AF is highly recommended with a rhythm control strategy to reduce the risk of AIC [7]. As medical therapy utilizing IV amiodarone infusion could not help in reverting our patient back to NSR while being symptomatic, electrocardioversion was guaranteed to reduce her mortality according to the most recent AHA guideline [8].

A study on 119 cases showed that ECV is a safe and effective procedure to be performed in the ED as no life-threatening arrhythmias or peripheral thromboembolism were recorded [9]. In contrast, Stiell et al. studied 1,736 cases who underwent ECV due to AF/flutter, despite high success rate (90.2%) of ECV in restoring NSR, seven patients had serious cardiac-related AE (hypotension requiring vasopressors or inotropes, bradycardia and sinus pause) [10]. Almost similar to our case, a patient with atrial flutter and incomplete RBBB received ECV that got complicated with sudden myocardial stunning leading to inevitable death [11].

Our patient had a prior history of rheumatic fever as a child and refractory atrial fibrillation that was unresponsive to pharmacological cardioversion, catheter ablation and even ECV. Given her acute presentation, her AF was likely complicated with AIC leading to a cumulative burden on her heart, making her susceptible to abrupt deterioration with a ECV. Based on her echocardiographic findings following ECV, myocardial stunning was highly suspected. Cardiac stunning has been defined as a prolonged mechanical myocardial dysfunction impairing ventricular or/and atrial activity which might occur due to different factors including postischemic ventricular dysfunction, vascular/endothelial injury, postischemic metabolic dysfunction or long-lasting impairment of neurotransmission with alterations in the heart electrophysiology [12]. In our reported case, following ECV, myocardial stunning emerged as a sudden global hypokinesis in the ventricles, and we hypothesize that it is likely due to a sudden alteration in the electrophysiological function of the cardiac myocytes after she received an long course of Amiodarone as well as her prior history of having ECV and catheter ablation which could have also contributed to the impaired cardiac neuromuscular activity. Other differentials of ventricular stunning following ECV is the transient increase in the QTc period as Younis et al. has reported nearly half of his participants underwent ECV and had QTc prolongation [13]. This did not apply to our case as her QTc got shorter from 410 to 397 msec. It is crucial to keep the possibility of unforeseen AE of ECV to provide immediate resuscitative support.

## Conclusion

Refractory cardiogenic shock due to ventricular stunning is a very rare complication after electrocardioversion and further research to identify patients at risk for this fatal complication is warranted.

## Learning Objectives

1. Sudden ventricular stunning one of the rare but possible complication following electrocardioversion in patients with AF leading to death.
2. Early identification and prompt treatment of such a complication might improve the outcomes.

## Electronic supplementary material

Below is the link to the electronic supplementary material.

Supplemental video 1: Parasternal long axis echocardiogram showing diffuse reduction in the wall motion of the left ventricle with an EF of 20%. Percutaneous heart pump is placed in the left ventricle passing the aortic valve

## Data Availability

The datasets used and/or analyzed during the current study available from the corresponding author on reasonable request.

## Declarations

### Event Timeline

2014: Diagnosed with atrial fibrillation, treated with metoprolol. June/2021: Patient developed tachycardia-induced cardiomyopathy, underwent electrocardioversion. Unsuccessful. July/2021: She underwent catheter ablation for recurrent atrial fibrillation, reverted to normal sinus rhythm. September/2021 (Day 1 of admission): She presented with sudden onset of dyspnea and palpitations, diagnosed with atrial fibrillation and rapid ventricular response requiring cardioversion. Initiated on pharmacological cardioversion and then advanced to electrical cardioversion. She decompensated and bedside TTE showed severe global hypokinesis with ventricular stunning. She was diagnosed with cardiogenic shock and placed on pressors. Day 2: Left and Right heart catheterization showed normal coronary arteries with moderate pulmonary hypertension, low CO/CI and elevated SVR confirming cardiogenic shock. Patient was placed on IABP. Day 3: Due to lack of hemodynamic improvement, patient was switched to Impella 2.5 instead of IABP. Patient was maximized on pressors. Day 4: Goals of care discussion with the family, they pursued comfort care measures and patient ultimately passed away.

## Abbreviations

AF: Atrial Fibrillation
HF: Heart Failure
ECV: Electrocardioversion
NSR: Normal sinus rhythm
AE: Adverse Events
LV: Left ventricular
EF: Ejection fraction
ED: emergency department
BP: Blood Pressure
HR: Heart Rate
bmp: beats per minute
RVR: Rapid Ventricular Response
EKG: electrocardiogram
AST: Aspartate Aminotransferase
ALT: Alanine transaminase
BNP: Brain Natriuretic Peptide
TTE: Transthoracic Echocardiogram
PHTN: Pulmonary artery hypertension
J: Joule
PEA: Pulseless Electrical Activity
CPR: Cardiopulmonary resuscitation
CO: Cardiac output
CI: Cardiac index
SVR: Systemic Vascular Resistance
AIC: Arrythmia-induced Cardiomyopathy
AHA: American Heart Association
RBBB: Right bundle branch block
